# Enhanced solar energy conversion in Au-doped, single-wall carbon nanotube-Si heterojunction cells

**DOI:** 10.1186/1556-276X-8-225

**Published:** 2013-05-10

**Authors:** Leifeng Chen, Hong He, Shijun Zhang, Chen Xu, Jianjiang Zhao, Shichao Zhao, Yuhong Mi, Deren Yang

**Affiliations:** 1 State Key Lab of Silicon Materials and Department of Materials Science and Engineering, Zhejiang University, Hangzhou 310027, People's Republic of China; 2 College of Materials and Environmental Engineering, Hangzhou Dianzi University, Hangzhou 310018, People's Republic of China; 3 Key Laboratory of Eco-Textiles, Ministry of Education, Jiangnan University, Wuxi 214122, Jiangsu, People's Republic of China

**Keywords:** Solar cell, Single-wall carbon nanotube, Chemical doping, Conductivity, Au nanoparticles, Plasmon resonance

## Abstract

The power conversion efficiency (PCE) of single-wall carbon nanotube (SCNT)/n-type crystalline silicon heterojunction photovoltaic devices is significantly improved by Au doping. It is found that the overall PCE was significantly increased to threefold. The efficiency enhancement of photovoltaic devices is mainly the improved electrical conductivity of SCNT by increasing the carrier concentration and the enhancing the absorbance of active layers by Au nanoparticles. The Au doping can lead to an increase of the open circuit voltage through adjusting the Fermi level of SCNT and then enhancing the built-in potential in the SCNT/n-Si junction. This fabrication is easy, cost-effective, and easily scaled up, which demonstrates that such Au-doped SCNT/Si cells possess promising potential in energy harvesting application.

## Background

Photovoltaic devices based on nanomaterials may be one kind of next-generation solar cells due to their potential tendency of high efficiency and low cost [1]. Among them, carbon nanotube (CNT), possessing one-dimensional nanoscale structure, high aspect ratios, large surface area [2], high mobility [3], and excellent optical and electronic properties, could be beneficial to exciton dissociation and charge carrier transport, which allow them to be useful in photovoltaic devices [4-8]. In recent years photovoltaic devices and photovoltaic conversion based on the heterojunctions of CNT and n-type silicon have been investigated [9-12]. In those devices, electron–hole pairs are generated in CNT under illumination and are separated at the heterojunctions. This means that the CNT acts as the active layer of the cells for exciton generation, charge collection, and transportation, while the heterojunction acts for charge dissociation. The conductivity and transparency of the single-wall carbon nanotube (SCNT) films are two important factors for fabricating the higher performance of SCNT/n-Si solar cell. Kozawa had found that the power conversion efficiency (PCE) strongly depended on the thickness of the SCNT network and showed a maximum value at the optimized thickness [13]. Li had found that photovoltaic conversion of SCNT/n-silicon heterojunctions could be greatly enhanced by improving the conductivity of SCNT [14]. Therefore, the efficiency of the solar cells for SCNT/n-Si is directly related to the property of SCNT film. Recently, doping in CNT has been employed to improve the performance of their cells [15-17]. Saini et al. also reported that the heterojunction of boron-doped CNT and n-type Si exhibited the improved property due to boron doping [18]. Bai et al. found that the efficiency of Si-SCNT solar cells is improved to 10% by H_2_O_2_ doping [19]. Furthermore, it was reported that higher performance SCNT-Si hybrid solar cells could be achieved by acid doping of the porous SCNT network [20]. It is believed that the doping of CNT and the reduced resistivity are in favor of the charge collection and prevention of carriers from recombination, so the PCE of the CNT-based solar cells can be enhanced.

In this paper, we prepared a SCNT film on a n-Si substrate by an electrophoretic method, and then doping the SCNT by a simple method in a HAuCl_4_·3H_2_O solution at room temperature [21,22], to improve the PCE as the result of improved conductivity and increased density of carriers. In this experiment, it was found that p-type doping due to Au could shift down the Fermi level and enhanced the work function of SCNT so that the open circuit voltage was increased. It was also found that the conversion efficiency of the Au-doped SCNT cells was significantly increased compared with that of pristine SCNT/n-Si cells.

## Methods

SCNT of 95% purity with an outer diameter of 1 to 2 nm and lengths of 1 to 3 μm were purchased from Chengdu Organic Chemicals Co. Ltd., Chinese Academy of Sciences, (Chengdu, Sichuan, China). In the experiments, 1 to 3 mg of SCNT were added into 50 ml of analytically pure isopropyl alcohol in which Mg(NO_3_)_2_·6H_2_O at a concentration of 1 × 10^−4^ M was dissolved. This solution was subjected to the high-power tip sonication for 2 h. A small part of the solution was diluted in 200 ml of isopropyl alcohol and then placed in a sonic bath for about 5 h to form SCNT electrophoresis suspension.

Constructing the homogeneous semitransparent SCNT network is the first step for fabricating SCNT/n-Si photovoltaic conversion cell. So SCNT film was prepared by the method of electrophoretic deposition (EDP) [23]. A piece of n-type silicon wafer (cathode) and a stainless-steel plate (an anode) were immersed into the SCNT electrophoresis suspension at room temperature. The two electrodes were kept in parallel with a gap of 1 cm. The deposition was carried out for 10 min by applying a constant DC voltage of 100 V. After the EDP and drying in air, the SCNT film on the Si wafer was put into a diluted nitric acid solution to remove possible surviving Mg(OH)_2_ on the surface.

**Figure 1:**
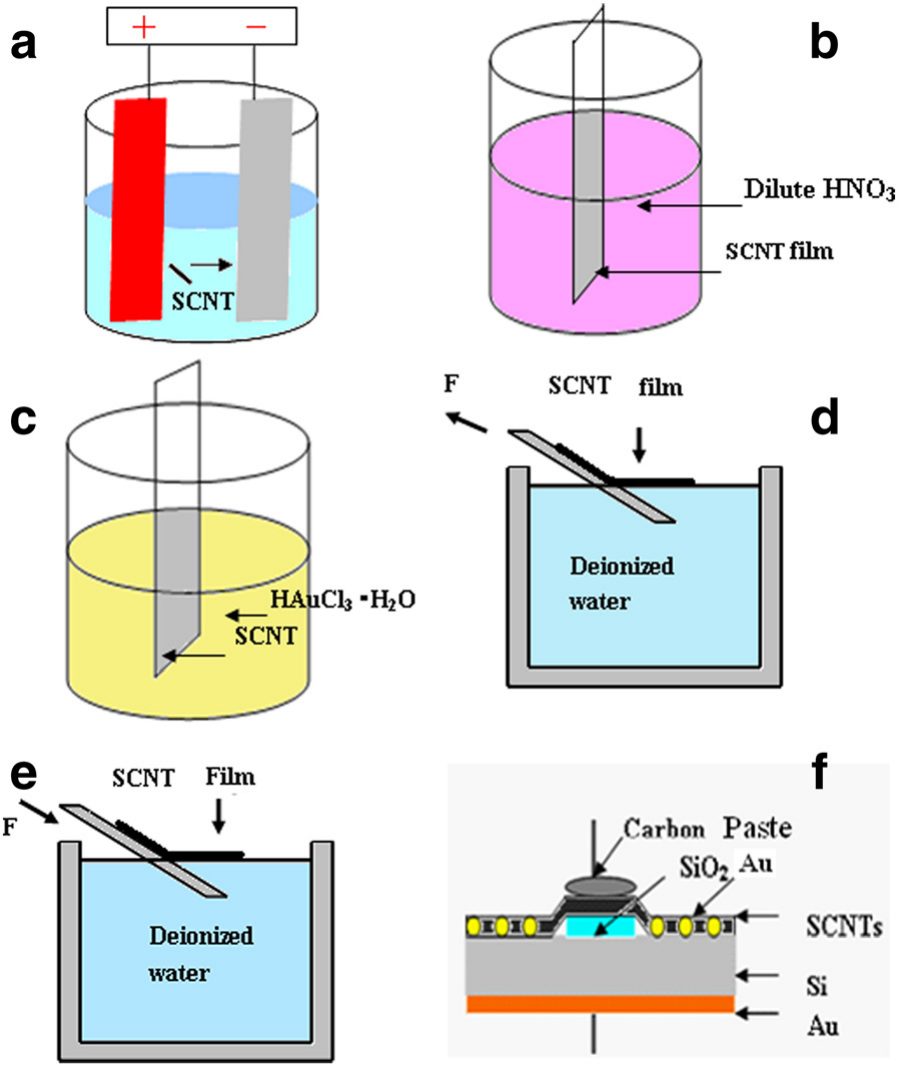
**Schematic diagrams of the EDP, doping and the configuration of a SCNT-on-silicon heterojunction solar cell.** (**a**) EDP SCNT film. (**b**) Removing Mg(OH)_2_ or Mg^+^ covered on the SCNT film in dilute nitric acid solution. (**c**) Doping the SCNT film in HAuCl_3_·H_2_O solution. (**d**) A Si substrate covered with SCNTs was slowly dipped into deionized water, and a SCNT film was peeled from the Si substrate and floated on water surface. (**e**) A patterned silicon wafer with a square SiO_2_ layer was used to pick up the SCNT film. (**f**) The configuration of a SCNT-on-silicon heterojunction solar cell.

The doping was carried out by means of dipping the SCNT film in a 0.3 mM hydrogen tetrachloroaurate(III) trihydrate (HAuCl_4_·3H_2_O) solution at different times. After drying in nitrogen atmosphere, the SCNT film was slowly dipped into deionized water. The SCNT film was peeled from the Si substrate and floated on the water surface. And then the n-type-patterned Si wafer with the thickness of 250 μm and the resistivity of 1 to 10 Ω·cm, which was pre-deposited with a square SiO_2_ layer of about 300 nm thickness, was immersed into the water to pick up the expanded SCNT films. Finally, the carbon paste was deposited on the SCNT films to form the upper electrode, and a layer of Au with the thickness of approximately 10 nm was deposited on the back side of the patterned Si wafer as the back electrode. The whole process of the heterojunction solar cells of SCNT and Si substrate is illustrated in Figure 1.

The morphology of SCNT network before and after doping was characterized by field emission scanning electronic microscope (FESEM) and transmission electronic microscope (TEM). The Raman spectra were measured with a laser Raman spectrophotometer. The excitation wavelength of the Ar ion laser was 514.5 nm. An ultraviolet–visible spectrometer (Varian Cary 100; Varian Inc., Palo Alto, CA, USA) was used to study the absorption of the SCNT film. The resistance of SCNT film was measured by a four-point probe method. The carrier density and mobility for the pristine SCNT film and doping film were measured with a Hall effect measurement system (Bio-Rad Corp. Hercules, CA, USA). An Oerlikon external quantum efficiency (EQE) measurement system (Oerlikon Co., Pfaffikon, Switzerland) was used to obtain the EQE of solar cells. The characteristics of cell performance were measured under the standard conditions (1 sun, AM 1.5 Global spectrum), using a Berger Flasher PSS 10 solar simulator (Berger Lichttechnik GmbH & Co. KG, Pullach im Isartal, Germany).

## Results and discussion

**Figure 2:**
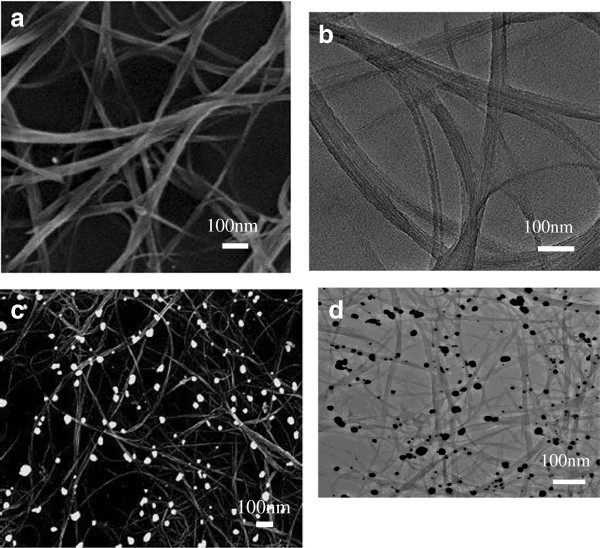
**SEM and TEM images of SCNT networks.** SEM (**a**, **c**) and TEM (**b**, **d**) images of SCNT networks fabricated by EDP and then Au doping.

From Figure 2a, it can be seen the porous network of SCNT were randomly distributed on the Si substrate. The networks of SCNT form the agglomerates of nanotube bundles containing many well-aligned tubes alternating with empty regions. In the Figure 2a, the TEM image shows that the SCNT film before doping is virtually free of catalyst residue. The SCNT film with thicknesses of 20–50 nm shows a transmission of more than 70% in the visible light region. Moreover, the SCNT lying on a substrate form numerous heterojunctions by contacting with the underlying n-Si. Such the semitransparent networks of SCNT ensure the solar light to arrive at interface of SCNT and the underlying Si wafer. After doping, Au nanoparticles with a size in the range of 20–80 nm cover on the surface of the SCNT, as seen in FESEM and TEM (inset) images in Figure 2c and Figure 2d.

**Figure 3:**
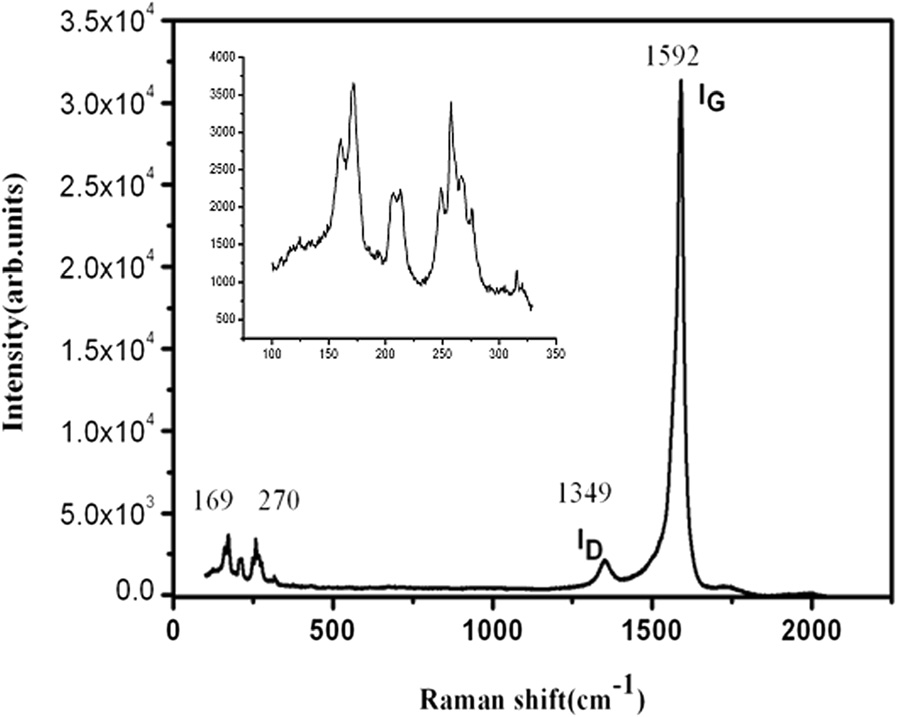
Raman spectra of the raw SCNT.

Figure 3 shows the Raman spectra of the commercial SCNT. It was obtained at room temperature with the laser wavelength of 514.5 nm. It can be seen from the spectra that the characteristic breath and tangential band of SCNT is at 169 to 270 cm^−1^ (inset) and 1,592 cm^−1^, respectively, while the characteristic peak of amorphous carbon is at 1.349 cm^−1^. In general, the content of *a-C* can be calculated by the following formula [24]

(1) $$\begin{array}{rl} {\left(I_{D}/I_{G}\right)}_{\mathrm{Commercial}\;\mathrm{SWCNT}}{}_{s} & =M_{a-C}\times {\left(I_{D}/I_{G}\right)}_{a-C}\\ & +M_{\mathrm{PureSCNTs}}\times {\left(I_{D}/I_{G}\right)}_{\mathrm{PureSCNT}}{}_{s} \end{array}$$

In formula (1), *M* means the molar ratio of the *a-C* and the SCNT, and *M*_*a-C*_ + *M*_pureSWCNTs_ =1, *I*_D_/*I*_G_ are the ratios of the intensities of D band and G band.

The *I*_D_*/I*_G_ value of commercial SCNT calculated from the Raman spectrum as shown in Figure 3 is about 0.70. Usually, the pure SCNT has very small *I*_D_*/I*_G_ value and could be assumed as 0.01 [24-26]. Meanwhile, the value of *I*_D_*/I*_G_ for *a-C* is similar to that of multiwall CNT (MCNT) and about 1.176 [24]. Thus, the calculated concentration ratio of amorphous carbon and SCNT is about 5.26%. It is obvious that the commercial SCNT is highly pure with little amorphous carbon.

**Figure 4:**
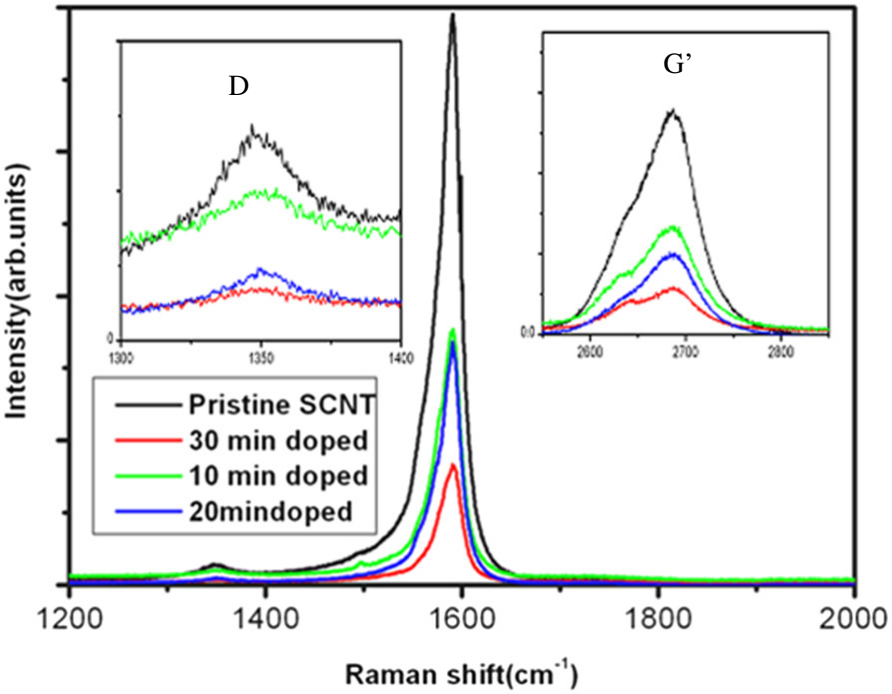
**Raman spectra of pristine and different doping time of SCNT.** The insets are the enlarged images of D and G′ band.

In order to further investigate the effect of Au doping on the properties of SCNT, the Raman spectra for different Au doping samples are shown in Figure 4. In Figure 4, the G bands were up-shift after doping. These changes were consistent with the previous report of the phonon stiffening effect by p-type doping [27,28]. The decreased intensities of the G′ bands manifested the reduction of metallicity of SCNT [29]. The *I*_D_*/I*_G_ values of SCNT for different doping time calculated from the Raman spectrum as shown in Figure 3 are almost about of 0.70, although the intensities of *I*_D_ and *I*_G_ were decreased. These results confirm that the integrity and tubular nature of SCNTs are well preserved during Au doping because of the only process of electrons transferring from SCNT to Au^3+^. This process cannot bring any defects for SCNT [30,31].

**Figure 5:**
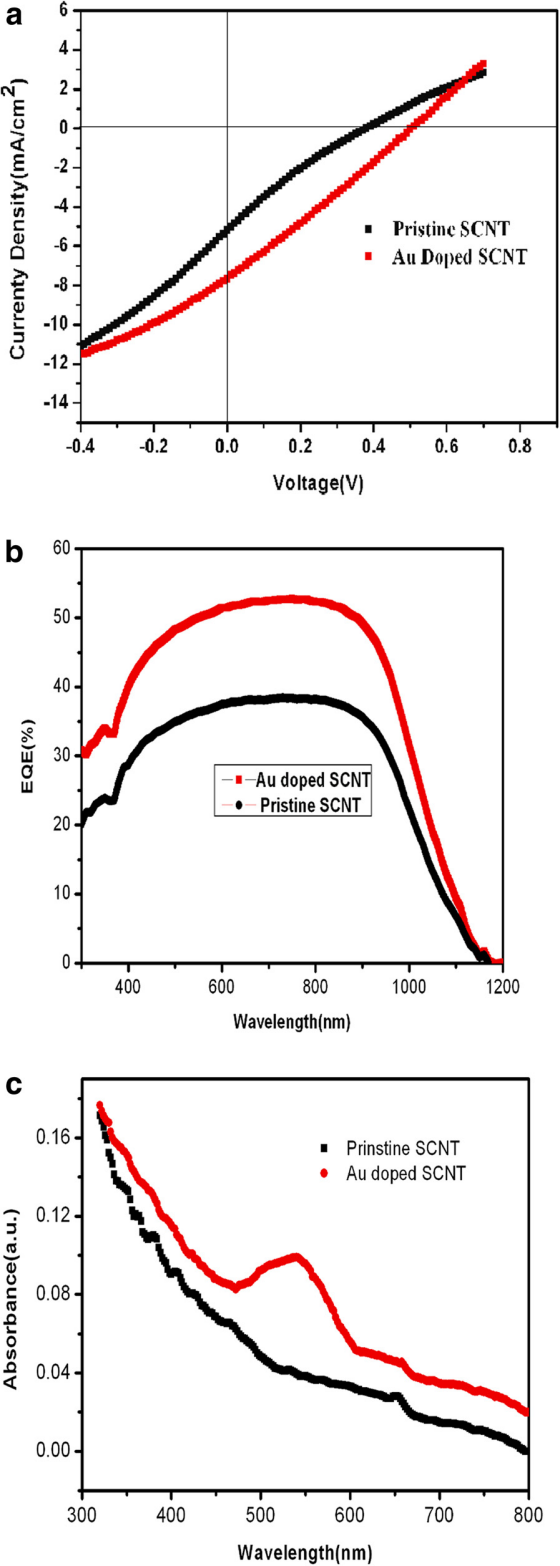
**Current–voltage characteristics, EQE of the solar cell, and optical absorption spectra of SCNT.** (**a**) Current–voltage characteristics of a typical SCNT/n-Si and Au-doped SCNT/n-Si heterojunction device. (**b**) The external quantum efficiency (EQE) of the solar cell obtained before (black line) and after (red line) Au doping. (**c**) Optical absorption spectra of SCNT before (black line) and after (red line) doping.

Figure 5a shows the current–voltage (*I-V*) curves of the solar cells before and after Au doping. Before doping, the cell exhibits an open circuit voltage (*V*_OC_) of 0.38 V, a *J*_SC_ of 5.20 mA/cm^2^, a fill factor (FF) of 0.18, and a PCE of 0.36%. After doping, the device shows *V*_OC_ of 0.50V, *J*_SC_ of 7.65 mA/cm^2^, FF of 0.30, and PCE of 1.15%. Both the *J*_SC_ and *V*_OC_ were enhanced after Au doping. The PCE was significantly increased to threefold. EQE results shown in Figure 5b indicate that after doping, the EQE increased in the measured spectral range from 300 to 1,200 nm [13,32-34]. The UV–vis spectrum of the Au nanoparticles (Figure 5c) shows a peak at about 535 nm, indicating the presence of a plasmon absorption band. The enhanced optical absorption was observed due to the increased electric field in the active photoactive layer by excited localized surface plasmons around the Au nanoparticles [35,36]. The EQE of the devices with the Au-doped SCNT is higher in the whole visible spectral range than that of the device with the SCNT. The enhanced EQE might be due to the increase of the conductivity of SCNT and of absorption by localized surface plasmons resonance.

In order to compare the SCNT network resistance before and after Au doping, we prepared the SCNT film (1 × 1 cm^2^) with parallel silver contacts on glass substrate. Four-probe measurements for the SCNT film showed that the sheet resistance can be reduced from 370 to 210 Ω/sq after Au doping. It is known that a standard oxidative purification process can induce p-type charge-transfer doping of SCNT which was observed in their field effect transistors [37]. In our experiments, the SEM and TEM images (the inset of Figure 2b) showed that Au nanoparticles formed during the electroless reduction of Au ions (Au^+3^) on the SCNT film. During the formation of Au nanoparticles on the SCNT surface, Au^+3^ played in the role of electron acceptors and received electrons from SCNT. The formation of Au particles on SCNT can be understood from an electrochemical perspective since the reduction potential of AuCl_4_^−^ ion is higher than the reduction potential of SCNT [38,39]. In aqueous solutions, the following reaction takes place on SCNT:

(2) $$\mathrm{AuC}l_{4}^{-}+3e\to Au^{0}↓+4Cl^{-}$$

**Figure 6:**
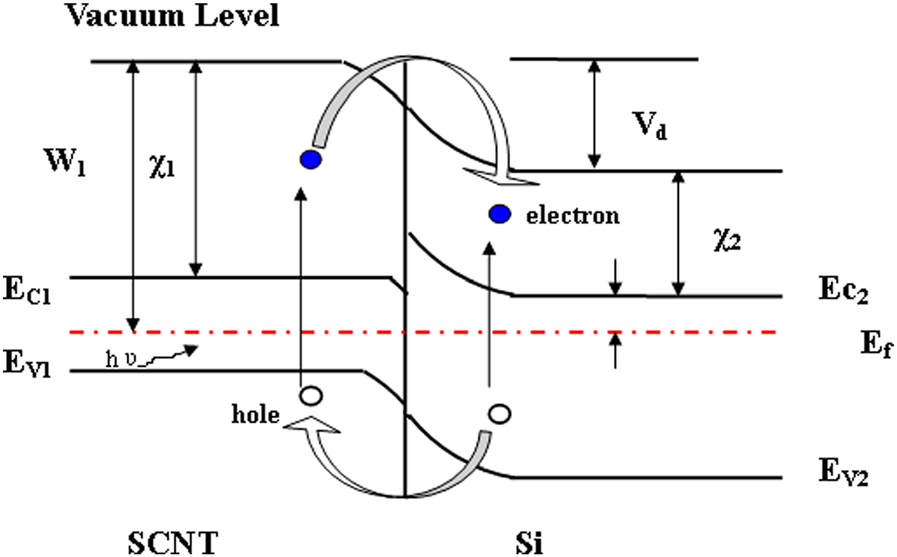
**Energy band diagram of the SCNT/n-Si heterojunction solar cell.** Dashed-dotted red line, hν; blue circle, electron.

As the electrons are depleted from the SCNT film, the hole carrier density increases, leading to the effective p-type doping effect [40-43]. Au doping can shift down the Femi level and enhance the work function of SCNT [44]; therefore, the built-in potential between SCNT and Si junction can be enhanced. As shown in Figure 6, the built-in voltage can be estimated by [20]

(3) $$V_{d}\approx \left(W_{1}-\chi_{2}-E_{C2}+E_{f}\right)/q$$

where *W*_1_ is the work function of SCNT, *χ*_2_ is the electron affinity of Si, (*E*_c2_ − *E*_f_) is the energy difference of conduction band and Femi level of n-type Si. Under illumination, the electrons and holes are generated in the SCNT film and the Si substrate. They are collected by the built-in voltage *V*_d_ at the junction, where holes and electrons are directed to the SCNT film and the n-Si substrate, respectively. Thus, the formation of the charge accumulation layer on both the sides can reduce the built-in potential, and the reduced potential is equal to the *V*_OC._ Thereby, the *V*_OC_ depends on the built-in potential height of the junction *V*_d_. Thus, the higher built-in potential height generates the higher *V*_OC_ under illumination, which can increase the power conversion efficiency of the cell.

In order to better understand the effect of Au doping on the carrier density and mobility of the SCNT, Hall effect measurements were performed for the SCNT film deposited on a glass substrate at room temperature. The Hall effect measurements revealed that the SCNT networks were all p-types conductivity before and after Au doping. After doping, an average carrier density for the SCNT film increased from 5.3 × 10^18^ to 1.4 × 10^20^ cm^−3^. This enhanced carrier density is advantageous for SCNT/n-Si photovoltaic devices because p doping and the reduced resistivity are in favor of charge collection and preventing carriers from recombination. The gold-hybridization SCNT can provide more charge transport paths, resulting in improved cell PCE more than three folds. Recent studies showed that doping also decreased the tunneling barrier between SCNT and concluded that this is the major fact in the overall film resistance [45-47]. So the devices series resistance (Rs) dropped from 218 Ω (or 8.72 Ω·cm^2^) in the SCNT/Si cell to 146 Ω (or 5.84 Ω·cm^2^) in the gold-hybridization SCNT-Si cell.

The effect of the immersion time of SCNT in HAuCl_4_·H_2_O solution on the photovoltaic characteristics of the device was investigated. The relative data are shown in the Table 1. It can be seen that with increasing immersion time, the PCE increases. But if the immersion time is too long, the efficiency of the device decreases, although the increasing absorbs of light increases (Figure 5b). Larger particles along with larger surface coverage lead to increased parasitic absorption and reflection, reducing the desired optical absorption in SCNT film layer [48]. In addition, the particles embedded between SCNT and Si substrate will reduce the density of p-n junction and lead to a significantly decrease shunt resistance; therefore, the *J*_SC_ and *P*_CE_ decrease. This means that too many Au nanoparticles and very large particles covering on the SCNT will reduce their device PCE.

**Table 1:** Photovoltaic characteristics of SCNTs-Si solar cell for SCNT immersion in Au solution at different times

**Time (min)**	***J***_**SC **_**(mA/cm**^**2**^**)**	***V***_**oc **_**(V)**	**FF (%)**	**PCE (%)**	**Rs (Ω cm**^**2**^**)**	**Carrier density (cm**^**−2**^**)**
0	5.2 ± 0.05	0.38 ± 0.02	18 ± 0.01	0.36 ± 0.06	8.72 ± 0.01	5.3 × 10^18^
10	7.2 ± 0.04	0.45 ± 0.01	26 ± 0.01	0.84 ± 0.04	7.5 ± 0.02	7.9 × 10^19^
20	7.65 ± 0.06	0.50 ± 0.02	30 ± 0.02	1.15 ± 0.05	5.84 ± 0.01	1.4 ×10^20^
30	7.46 ± 0.05	0.47 ± 0.01	31 ± 0.01	1.09 ± 0.04	5.65 ± 0.02	1.3 × 10^21^
40	7.1 ± 0.02	0.46 ± 0.02	30 ± 0.01	0.98 ± 0.01	5.63 ± 0.02	1.5 × 10^21^

## Conclusions

In summary, the photovoltaic performance of SCNT-Si heterojunction devices can be significantly improved by doping Au nanoparticles on the wall of SCNT. In the experiments, the PCE, open circuit voltage, short-circuit current density, and fill factor of the devices reached to 1.15%, 0.50 V, 7.65 mA/cm^2^, and 30% from 0.36%, 0.38v, 5.2, and 18%, respectively. The improved conductivity and the enhanced absorbance of active layers by Au nanoparticles are mainly the reasons for the enhancement of the PCE. It is believed that the photovoltaic conversion efficiency can be further improved by optimizing some factors, such as the density of SCNT, the size and shape of Au nanoparticles, and efficient electrode design.

## Competing interests

The authors declare that they have no competing interests.

## Authors’ contributions

LC carried out the total experiment, participated in the statistical analysis, and drafted the manuscript. HH, SZ, and CX carried out part of the experiments. JZ and YM participated in the guidance of the experiment. SZ and LC conceived of the study and participated in its design and coordination. DY guided the revision of the manuscript. All authors read and approved the final manuscript.
